# Supplementary material for: Novel Parkinson’s Disease Genetic Risk Factors Within and Across European Populations
Source: medRxiv. 2025 Mar 17:2025.03.14.24319455. Preprint. [Version 1] doi: 10.1101/2025.03.14.24319455 (PMC11957085; doi:10.1101/2025.03.14.24319455)
Supplement: 1 [file NIHPP2025.03.14.24319455V1-supplement-1.pdf]

medRxiv preprint doi: https://doi.org/10.1101/2025.03.14.24319455; this version posted March 17, 2025. The copyright holder for this preprint (which was not certified by peer review) is the author/funder, who has granted medRxiv a license to display the preprint in perpetuity. This article is a US Government work. It is not subject to copyright under 17 USC 105 and is also made available for use under a CC0 license.

SUPPLEMENTARY MATERIALS

Figure S1: Beta-beta plot comparing effects from Nalls et al 2019 and the new joint 2025 meta-analysis for the 90 lead SNPs from Nalls et al 2019

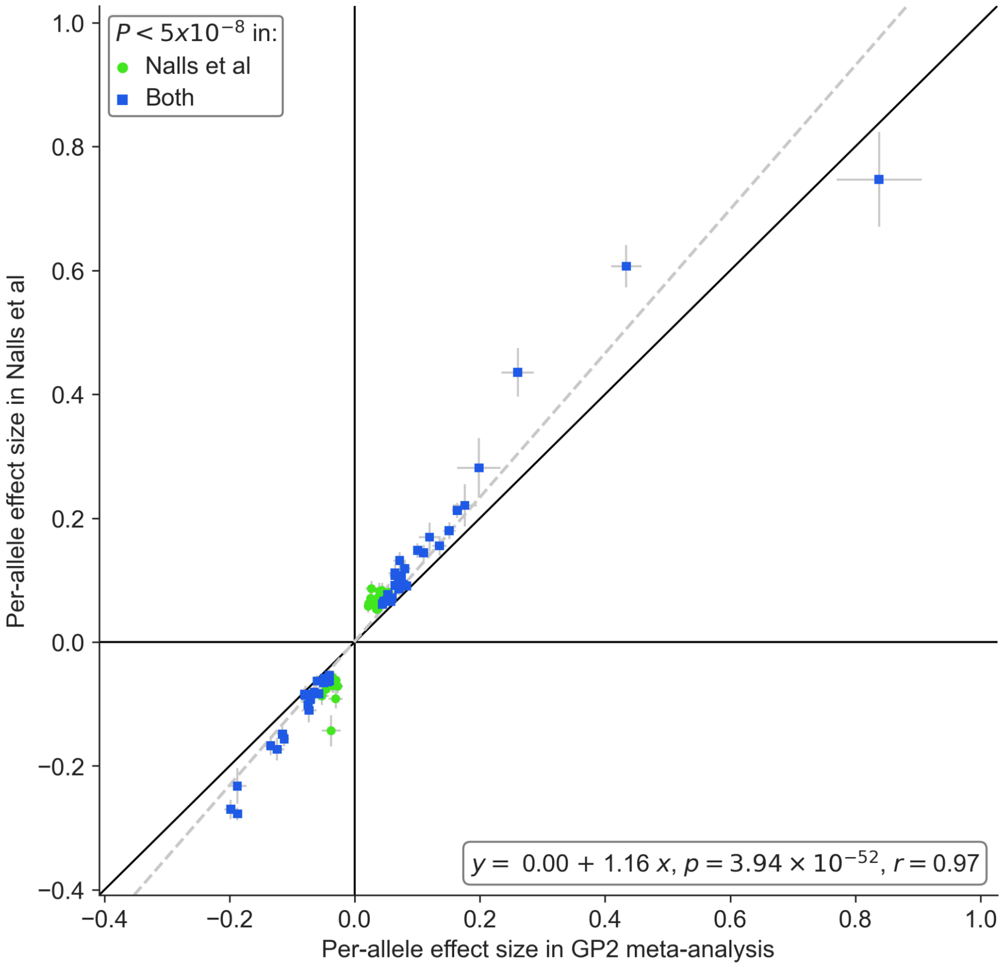

Tables

Supplementary Tables

Appendix: Local association plots and forest plots for all loci.
